# Limitations of fexofenadine limited sampling strategy using plasma concentrations and partial area under the concentration-time curve to estimate transporter activity in healthy adults

**DOI:** 10.5414/CP204380

**Published:** 2023-04-12

**Authors:** Marlon Liyanage, Chelsea L. Blaquera, Joseph Piscitelli, Scott R. Penzak, Thomas D. Nolin, Mary F. Paine, Joseph D. Ma

**Affiliations:** 1 Skaggs School of Pharmacy and Pharmaceutical Sciences, University of California San Diego, La Jolla, CA,; 2 Harrison School of Pharmacy, Auburn University, Auburn, AL,; 3 University of Pittsburgh School of Pharmacy, Pittsburgh, PA, and; 4 College of Pharmacy & Pharmaceutical Sciences, Washington State University, Spokane, WA, USA

**Keywords:** drug-drug interaction, fexofenadine, limited sampling strategy, transporter

## Abstract

Objective: Fexofenadine is a probe drug used to phenotype P-glycoprotein (P-gp) and organic anion transporting polypeptide (OATP) 1B1/3 activities. This study evaluated a limited sampling strategy using plasma concentrations and/or partial area under the concentration versus time curves (AUCs) to estimate systemic exposure and, potentially, P-gp and OATP1B1/3 activities. Materials and methods: Plasma concentration versus time data were obtained from 53 healthy adult participants (22 females) from four published studies. Participants were administered a single oral dose (120 mg) of fexofenadine during constitutive P-gp and OATP1B1/3 conditions. Concentration-time data were divided into a training (n = 18) and validation (n = 35) set. Backwards stepwise linear regression generated single-, 2-timepoint, and partial AUC limited sampling models (LSMs). Noncompartmental analysis methods were used to determine total AUC (AUC_0–lNF_) from intensive sampling. Coefficient of determination (r^2^) and bias and precision were assessed via relative percent mean prediction error (%MPE), relative percent mean absolute error (%MAE), and relative percent root mean square error (%RMSE). Results: The geometric mean observed AUC_0–INF_ was 1,680 ng×h/mL. The 2-, 5-, and 2- plus 5-hour LSMs met backwards stepwise linear regression significance (p < 0.15) to remain in the model but had unacceptable %RMSE (17 – 29%). The majority of partial AUC LSMs had unacceptable r^2^ (0.21 – 0.83), with all models having unacceptable %MAE (12 – 35%). Conclusion: Fexofenadine limited sampling strategy using single-timepoint, 2-timepoint, and partial AUCs were unable to accurately estimate AUC_0–lNF_ and thus constitutive P-gp and OATB1B1/3 activities in healthy adults. Timepoints that were not measured or selected may have improved LSM performance.

Presented in part at the American Society for Clinical Pharmacology & Therapeutics Annual Meeting, March 2022 and American College of Clinical Pharmacology Annual Meeting, September 2022. 

**What is known about this subject**

- Fexofenadine is a probe drug used to phenotype P-glycoprotein (P-gp) and organic anion transporting polypeptide (OATP) 1B1/3 activities. Limited sampling strategy is a validated method that estimates exposure from a reduced number of blood or plasma samples. The application of limited sampling strategy has extended to metabolism-mediated drug-drug interaction studies. Studies have suggested a fexofenadine single-timepoint, as well as several 2-timepoint, limited sampling models to estimate exposure.

**What this study adds**

- Limited sampling strategy using fexofenadine single-timepoint and 2-timepoint concentrations did not accurately estimate exposure_. _
- Limited sampling strategy using fexofenadine partial AUCs did not accurately estimate exposure.
- We do not recommend fexofenadine limited sampling strategy to assess constitutive P-gp and OATB1B1/3 activities in healthy adults.

## Introduction 

Phenotyping with respect to pharmacokinetic drug-drug interactions (DDIs) refers to the quantification of in vivo drug metabolizing enzyme (e.g., cytochrome P450 (CYP)) and transporter activities, results from which can be used to assess DDI risk. Fexofenadine is one probe drug recommended by the U.S. Food and Drug Administration that is used to phenotype the efflux transporter P-glycoprotein (P-gp) and uptake transporters organic anion transporting polypeptide (OATP) 1B1 and OATP1B3 [1]. Perturbations in fexofenadine area under the plasma concentration versus time curve (AUC) and/or systemic or apparent oral clearance are assumed to reflect P-gp and/or OATP1B1/3 activities [2, 3]. Generally, determination of an AUC requires intense sampling, with the last collection occurring at 3 times the elimination half-life of the drug or later. Regarding fexofenadine (elimination half-life, 11 – 14 hours), the last collection should be at minimum ~ 42 hours or later [4, 5]. 

Limited sampling strategy is a validated method that estimates AUC and/or systemic or apparent oral clearance from a reduced number of blood or plasma samples. In certain populations, such as geriatric, pediatric, and/or cancer patients, intense sampling is difficult due to collection frequency and duration, venipuncture access, and volume collection limits. Thus, such an approach is advantageous in alleviating the inconvenience and cost of intense sampling. Limited sampling strategy has been validated for several immunosuppressants (e.g., mycophenolic acid, tacrolimus, and cyclosporine), with the potential for clinical use in kidney and liver transplant patients [6, 7, 8, 9]. There is also evidence that limited sampling strategy has utility for bioequivalence studies for drugs with a prolonged elimination half-life [10]. 

The application of limited sampling strategy has extended to metabolism-mediated DDI studies, with the majority evaluating midazolam, an established phenotyping probe drug for CYP3A. Limited sampling models (LSMs) of single-, 2-, and 3-timepoint metabolic ratios of 1’-hydroxymidazolam to midazolam and partial AUCs have been evaluated with conflicting results [11, 12, 13, 14, 15]. Chaobal and Kharash [11] recommended an optimal midazolam concentration timepoint of 5 or 6 hours that encompassed a broad spectrum of CYP3A enzyme activity, including constitutive, inhibited, and induced conditions. Other studies do not support single-, but rather 2- and/or 3-timepoint midazolam LSMs to accurately estimate CYP3A activity [15, 16]. Regarding transporter-mediated DDI studies using fexofenadine as a probe drug, 2 studies suggested a single-timepoint at maximum concentration (C_MAX_) and several 2-timepoint (1.5 plus 4 hour, 2 plus 4 hour) LSMs to estimate transporter activity [2, 3]. 

Based on these observations, the objectives of this study were two-fold. The first was to determine if the fexofenadine LSM at the 2 plus 4 hour timepoints was reproducible using different concentration-time data from which the LSMs were derived. This key validation step would permit the utilization of pre-specified timepoint LSMs for general use in future transporter-mediated DDI studies. The second objective was to determine novel LSMs using partial AUCs as possible alternative phenotypic metrics to estimate transporter activity. The rationale was based on published studies that examined partial AUCs to estimate midazolam metabolic clearance as well as bioequivalence studies [12, 17]. 

## Materials and methods 

### Study participants 

Study exempt status was granted by the University of California, San Diego Human Research Protections Program (201959XX). Fexofenadine plasma concentration-time data from four previous studies were obtained (Table 1) [5, 18, 19, 20]. A total of 53 healthy adults participated in these studies. Demographic data were provided for most of the studies (Table 1). Individuals who were at least 18 years of age, able to provide written informed consent, had no clinically significant medical history and/or physical examination findings, and willing to abstain from any medications and/or dietary supplements and other natural products for at least 7 days prior to the study were eligible to participate. Exclusion criteria included the presence of clinically significant (greater than 2 times the upper normal limits) blood and urine laboratory tests, current tobacco users, a history of adverse reaction(s) to fexofenadine, and individuals who were pregnant or breastfeeding. Healthy status was defined by assessment of medical history, physical examination, and routine blood and urine laboratory tests. Participants of child-bearing potential underwent a serum pregnancy test. All participants were administered a single oral dose (120 mg) of fexofenadine under constitutive P-gp and OATP1B1/3 conditions. 

### Sample collection and assay 

Plasma samples [2, 5, 18, 19, 20] were collected from each participant at various study-specific time points (Table 1). Plasma fexofenadine concentrations were measured using ultra-performance or high-performance liquid chromatography–mass spectrometry. The lower limit of quantification ranged from 0.1 to 1.0 ng/mL, with inter- and intra-assay precisions and accuracies < 15%. Details of each method are provided elsewhere [5, 18, 19, 20]. 

### Data analysis 

Noncompartmental analysis (Phoenix WinNonlin v 8.1) of fexofenadine concentration-time data was used to determine AUC_0–INF_ and partial AUCs. The linear trapezoidal method was used, and software-determined best fit was used for elimination phase determination. Fexofenadine concentrations and partial AUCs were log-transformed prior to backwards stepwise linear regression analyses. 

A limited sampling strategy was performed, which entailed randomizing participant concentration-time data into a training (n = 18) and validation (n = 35) set. LSMs were first determined using fexofenadine concentrations (independent variable) to estimate AUC from time zero to infinity (AUC_0–INF_, dependent variable). Post-dose timepoints common across all studies were used (e.g., 1, 2, 3, 4, 5, 6, and 8 hours) (Table 1). Backwards stepwise linear regression analyses were performed to estimate AUC_0–INF_ as a function of log-transformed fexofenadine concentrations from the training set (SAS version 9.3). Preset criteria for LSM selection were a significance level of 0.15 for model entry, a significance level of 0.05 for concentrations to remain in the model, and a coefficient of determination (r^2^) greater than or equal to 0.9. The LSMs were used to estimate AUC_0–INF_ from the validation set. 

This process was repeated to determine novel LSMs using different independent variables (partial AUCs) to estimate AUC_0–INF_. Selection of partial AUCs was based on previous studies involving midazolam and digoxin (a P-gp probe drug) [12, 21]. Backwards stepwise linear regression analyses were performed to estimate AUC_0–INF_ as a function of fexofenadine partial AUCs from the training set. The same preset criteria were used for LSM selection, with the LSMs subsequently estimating AUC_0–INF_ from the validation set. Bias and precision were assessed via relative percent mean prediction error (%MPE), relative percent mean absolute error (%MAE), and relative percent root mean square error (%RMSE). Acceptable limits were a %MPE of –5 to +5%, %MAE ≤ 10%, and %RMSE ≤ 15% [22]. 

## Results 

During constitutive P-gp and OATP1B1/3 conditions, the geometric mean observed AUC_0–INF_ was 1,680 ng×h/mL. Single-timepoint LSMs at 1, 3, 4, 6, and 8 hours, as well as 2-timepoint LSMs at 1 plus 2 hours, 1 plus 4 hours, 1 plus 8 hours, 2 plus 4 hours, 2 plus 8 hours, and 4 plus 8 hours did not meet backwards stepwise linear regression significance for model entry and/or to remain in the model. The 2 hour and the 5 hour single-timepoint LSMs met significance criteria but had unacceptable r^2^, %MAE, and %RMSE (Table 2). Although the 2- plus 5-hour timepoint LSM had an acceptable r^2^ and %MPE, unacceptable %MAE and %RMSE were observed (Table 2). 

Fexofenadine partial AUC LSMs are summarized in Table 3. The majority of partial AUC LSMs overestimated AUC_0–INF_. In contrast, the partial AUC_4–8_ and partial AUC_6–8_ underestimated AUC_0–lNF_. The majority of partial AUC LSMs had unacceptable r^2^ and %RMSE, with the exception of the partial AUC_2–6_ LSM (r^2^ = 0.91, %RMSE = 14%). Partial AUC_2–6_, AUC_4–8_, and AUC_6–8_ LSMs had an acceptable %MPE of 2%, –5%, and –5%, respectively. All evaluated partial AUC LSMs had an unacceptable %MAE (Table 3). 

## Discussion 

The first objective of this study was to confirm whether a fexofenadine LSM at 2 plus 4 hours was reproducible. The impetus for this confirmatory step was based on limited sampling strategy studies involving oral midazolam. Lee et al. [23] recommended a 2- and a 3-timepoint LSM to estimate oral midazolam systemic exposure. The same LSMs were then applied using external data (n = 106) from six previously published studies during CYP3A constitutive, inhibition, and induction/activation conditions [24]. During constitutive CYP3A conditions, the 2- and 3-timepoint LSMs accurately estimated exposure for 2 of 6 and 3 of 6 studies, respectively. In contrast, the 2- and 3-timepoint LSMs were unable to estimate AUC during CYP3A inhibition conditions for 0 of 4 and 3 of 4 studies, respectively. These results remained consistent during CYP3A induction/activation conditions [24]. Consequently, the contrasting results between each study during various CYP3A conditions limit the widespread utility of these midazolam LSMs [23]. Similar findings with intravenous midazolam and oral S-warfarin (a CYP2C9 probe drug) LSMs are published elsewhere [25, 26]. 

This study was unable to confirm the accuracy of the fexofenadine LSMs at 2 plus 4 hours and 1.5 plus 4 hours. Because the 1.5 hour timepoint was not collected across all studies, the 1.5 plus 4 hour LSM was not evaluated. During backwards stepwise linear regression analyses, a 2 plus 4 hour LSM was generated but did not meet significance levels for model entry. These results contrast with those from a previous study [2]. One difference between studies was the acceptance limits for r^2^, %MPE, and %MAE. Less conservative acceptance limits would be appropriate for scenarios where increased fexofenadine pharmacokinetic variability is anticipated, such as during conditions of transporter inhibition or in select individuals with altered P-gp and/or OATP1B1/3 function. Conservative acceptance limits used in the current study were chosen due to the anticipated decreased variability in fexofenadine systemic exposure in healthy adult participants. If the same acceptance limits were applied to the aforementioned 2 plus 4 hour LSM, an unacceptable r^2^ (0.88) would result [2]. 

Another difference between studies was the fexofenadine dose. Coelho et al. [2] selected a subtherapeutic dose (10 mg), which was less than one-tenth the approved therapeutic dose used in the current study (120 mg). The lower dose was selected presumably to enhance the safety window of fexofenadine. However, additional research is needed to confirm the utility of this dose. Evidence of dose proportionality at 10 mg is lacking, as 1 study reported dose proportionality from 20 to 240 mg [27]. In the context of microdosing, as well as subtherapeutic doses, linear pharmacokinetics are required for accurate estimation of transporter activity. Additionally, fexofenadine LSMs may only be suitable using the 10-mg dose due to site-specific effects, whereby the observed LSMs may only be applicable to the participants and conditions from which the models were developed [28]. 

One strength of the current study was the appropriate validation of fexofenadine LSMs by evaluating bias (%MPE) and precision (%MAE, %RMSE). Bias represents systematic error and was observed by the over- or under-estimation of fexofenadine exposure (Table 3). Precision is random error and represents the variable magnitude in the estimation. Another study recommended the fexofenadine C_max_ to estimate AUC_INF_ based on a strong correlation (r = 0.97) [3]. The term r only provides information about an association, not bias and precision. Using correlations to validate LSMs is not appropriate. Thus, caution is warranted using the fexofenadine C_max_ LSM. 

The second objective of this study was to identify novel LSMs using partial AUCs as possible alternative phenotypic metrics to estimate transporter activity. All evaluated partial AUC LSMs had unacceptable %MAEs, with the majority of them overestimating AUC_0–INF_. One possible reason the partial AUCs were not suitable are distribution effects early in the dosing interval, thus impacting partial AUCs beginning at time 0 to 1 hour. Partial AUC_2–4_ and AUC_2–6_ improved but still overestimated AUC_0–INF_ (Table 3). Another possibility is that partial AUC LSMs are probe drug specific, with no clear consensus about the optimal partial AUC to recommend. In studies involving oral digoxin and oral midazolam, the recommended partial AUCs were AUC_0–4_ and AUC_2–4_ (exclusively during constitutive CYP3A conditions), respectively [12, 21]. 

Addition of a third concentration timepoint and/or expansion of the time period beyond 5 hours for partial AUCs may have improved LSM bias and precision. In a study evaluating multiple LSMs for estimating cyclosporine AUC, the largest reduction in bias, assessed via prediction error, was between single- and 2-timepoint LSMs. There was minimal improvement in bias between 2- and 3-timepoint LSMs. Additionally, a cyclosporine 3-timepoint LSM produced the same %MPE that was beyond acceptable limits compared to the 2-timepoint LSM [8]. Thus, adding more independent variables via additional timepoints and/or partial AUCs with an expanded time period may not result in improved bias and/or precision. The number of blood or plasma samples to be included in LSMs remains an unanswered question but depends on the extent of interindividual pharmacokinetic variability of the probe drug and timing of sample collection. 

Although the fexofenadine concentrations were measured using liquid chromatography-tandem mass spectrometry, differences in analytical specificity and techniques between study sites may have impacted the accuracy and utility of a partial AUC approach. For example, midazolam concentration data (n = 73) were obtained from different institutions using different analytical methods [16]. Regardless of the method, several midazolam LSMs were generated, and each LSM had acceptable bias and precision [16]. Finally, the study participants were not genotyped for *MDR1* and *OATB1B1* genetic polymorphisms that contribute to fexofenadine pharmacokinetic variability, with some polymorphisms varying among ethnic groups [29]. However, the impact of ethnicity on fexofenadine pharmacokinetics may be limited as interindividual variability within a group is comparable to interethnic variability between groups [30]. 

In conclusion, the evaluated fexofenadine single-timepoint, 2-timepoint, and partial AUC LSMs were unable to accurately estimate fexofenadine AUC in healthy adult participants. Consequently, the evaluated fexofenadine LSMs could not be used to estimate P-gp and OATP1B1/3 activities. Other P-gp and/or OATB1B1/3 probe drugs include dabigatran etexilate, digoxin, and rosuvastatin, and a limited sampling approach for each is under evaluation. 

## Authors’ contributions 

Joseph D. Ma conceived of and designed the study. Scott R. Penzak, Thomas D. Nolin, and Mary F. Paine provided fexofenadine concentration data. Marlon Liyanage, Chelsea L. Blaquera, Joseph Piscitelli, and Joseph D. Ma performed data collection, pharmacokinetic analysis, and statistical analysis. Marlon Liyanage, Chelsea L. Blaquera, and Joseph D. Ma composed a manuscript draft. All authors reviewed versions and provided revisions of multiple draft manuscripts. 

## Funding 

Marlon Liyanage and Joseph Piscitelli are post-doctoral fellows with funding supported by the UC San Diego Skaggs School of Pharmacy and Pharmaceutical Sciences (SSPPS) and Pfizer, Inc. Chelsea L. Blaquera received funding support from the Skaggs Scholarship from UC San Diego, SSPPS. Funding support was provided by the National Center for Complementary and Integrative Health grant (U54 AT008909 to Mary F. Paine). 

## Conflict of interest 

All authors declare no conflict of interest. 

**Table 1.** Summary of published oral fexofenadine studies for data analysis.

Study	Number of participants^a^	Age (range, years)	Weight (range, kg)	Fexofenadine collection timepoints (h)	Reference
1	10 (4 females)	25 – 59	64 – 97	0, 0.5, **1**, **2**, **3**, **4**, **5**, **6**, **8**, 12	[19]
2	18 (9 females)	23 – 60	NA	0, **1**, 1.5, **2**, **3**, **4**, **5**, **6**, **8**, 10, 12, 24, 36, 48	[5]
3	12 (4 females)	23 – 42	51 – 114	0, 0.5, **1**, 1.5, **2**, 2.5, **3**, 3.5, **4**, **5**, **6**, **8**, 24	[18]
4	13 (5 females)	26 – 45	51 – 94	0, 0.5, **1**, 1.5, **2**, 2.5, **3**, 3.5, **4**, **5**, **6**, **8**, 24	[20]

^a^Healthy adult participants were administered a single oral dose (120 mg) of fexofenadine. Bold values indicate common timepoints among the four studies. NA = not available.

**Table 2.** Limited sampling models of fexofenadine concentration(s) to estimate exposure and assessment of bias and precision during constitutive P-gp and OATP1B1/3 conditions.

Limited sampling model^a^	Estimated AUC_0–lNF_^b^	r^2^	%MPE	%MAE	%RMSE
0.49 × logC_2_ + 2.12	1,990	0.64	11%	24%	29%
0.96 × logC_5_ + 1.15	1,550	0.86	**–5%**	18%	22%
0.22 × logC_2_ + 0.73 × logC_5_ + 1.15	1,640	**0.94**	**–1%**	14%	17%
Acceptable criteria		≥ 0.9	–5 to 5%	≤ 10%	≤ 15%

^a^Limited sampling models were derived from training set data (n = 18), ^b^Expressed as geometric mean values; units are ng×h/mL. Bold values indicate the acceptance criterion was met. AUC_0–INF_ = area under the concentration versus time curve from time 0 to infinity; logC_X_ = log transformed (base 10) concentration at timepoint X; r^2^ = coefficient of determination; %MAE = relative percent mean absolute error; %MPE = relative percent mean prediction error; %RMSE = relative percent root mean square error.

**Table 3.** Limited sampling models of fexofenadine partial AUC to estimate exposure and assessment of bias and precision during constitutive P-gp and OATP1B1/3 conditions.

Limited sampling model^a^	Estimated AUC_0–INF_^b^	r^2^	%MPE	%MAE	%RMSE
0.20 × logAUC_0–1_ + 2.98	2,180	0.21	19%	35%	43%
0.47 × logAUC_0–2_ + 2.19	2,110	0.52	19%	30%	38%
0.70 × logAUC_0–4_ + 1.3	1,970	0.78	13%	20%	24%
0.77 × logAUC_0–5_ + 1.02	1,910	0.83	10%	17%	20%
0.49 × logAUC_1–2_ + 2.2	`2,070	0.56	16%	27%	34%
0.69 × logAUC_1–4_ + 1.36	1,930	0.78	10%	18%	22%
0.72 × logAUC_2–4_ + 1.39	1,860	0.81	7%	16%	19%
0.89 × logAUC_2–6_ + 0.71	1,730	**0.91**	**2%**	12%	**14%**
0.93 × logAUC_4–8_ + 0.76	1,580	0.83	**–5%**	15%	18%
0.83 × logAUC_6–8_ + 1.4	1,630	0.68	**–5%**	16%	20%
Acceptable criteria		≥ 0.9	–5 to 5%	≤ 10%	≤ 15%

^a^Limited sampling models were derived from training set data (n = 18); ^b^Expressed as geometric mean values; units are ng×h/mL. Bold values indicate the acceptance criterion was met. AUC_0–INF_ `= area under the concentration time curve from time 0 to infinity; logAUC_X–Y_ = log transformed (base 10) partial area under the concentration time curve from time X to Y; r^2^ = coefficient of determination; %MAE = relative percent mean absolute error; %MPE = relative percent mean prediction error, %RMSE = relative percent root mean square error.
